# Health-seeking behavior of COVID-19 cases during the first eight weeks of the outbreak in Singapore: differences between local community and imported cases and having visits to single or multiple healthcare providers

**DOI:** 10.1186/s12889-022-12637-8

**Published:** 2022-02-05

**Authors:** Min Zhi Tay, Li Wei Ang, Wycliffe Enli Wei, Vernon J. M. Lee, Yee-Sin Leo, Matthias Paul H. S. Toh

**Affiliations:** 1 National Public Health and Epidemiology Unit, National Centre for Infectious Diseases, 16 Jalan Tan Tock Seng, Singapore, 308442 Singapore; 2 Ministry of Health, Singapore, Singapore; 3 Saw Swee Hock School of Public Health, Singapore, Singapore; 4 Tan Tock Seng Hospital, Singapore, Singapore; 5 Lee Kong Chian School of Medicine, Singapore, Singapore; 6 Yong Loo Lin School of Medicine, Singapore, Singapore; 7 National Centre for Infectious Diseases, Singapore, Singapore

**Keywords:** Primary Care, Coronavirus, Outbreaks, Epidemiology, Health Behavior

## Abstract

**Background:**

COVID-19 is a novel pandemic affecting almost all countries leading to lockdowns worldwide. In Singapore, locally-acquired cases emerged after the first wave of imported cases, and these two groups of cases may have different health-seeking behavior affecting disease transmission. We investigated differences in health-seeking behavior between locally-acquired cases and imported cases, and within the locally-acquired cases, those who saw single versus multiple healthcare providers.

**Methods:**

We conducted a retrospective study of 258 patients who were diagnosed with COVID-19 from 23 January to 17 March 2020. Variables related to health-seeking behavior included number of visits prior to hospitalization, timing of the first visit, duration from symptom onset to admission, and places where the cases had at least one visit.

**Results:**

Locally-acquired cases had longer duration from onset of symptoms to hospital admission (median 6 days, interquartile range [IQR] 4–9) than imported cases (median 4 days, IQR 2–7) (*p* < 0.001). Singapore residents were more likely to have at least one visit to private clinics and/or government-subsidized public clinics than non-residents (84.0% vs. 58.7%, *p* < 0.001). Among locally-acquired cases, those who sought care from a single healthcare provider had fewer visits before their hospital admissions compared with those who went to multiple providers (median 2 vs. 3, *p* = 0.001).

**Conclusion:**

Our study indicates the need to encourage individuals to seek medical attention early on in their patient journey, particularly from the same healthcare provider. This in turn, would facilitate early detection and isolation, hence limiting local transmission and enabling better control of the COVID-19 outbreak.

## Background

Nearly two years have passed since the severe acute respiratory syndrome coronavirus 2 (SARS-CoV-2) was declared by the World Health Organization as a pandemic. An intact and comprehensive surveillance system remains one of the most important public health measures in this prolonged pandemic with over 255 million cases reported worldwide as of 19 November 2021 [1]. In Singapore, a total of 1,375 cases had been reported as of 7 April 2020 since the start of the outbreak [2], prior to the implementation of an elevated set of safe distancing measures, as a “circuit breaker” to pre-empt the trend of increasing local transmission of COVID-19. This was one of the measures Singapore swiftly adopted in order to “flatten the epidemic curve” and avoid overwhelming the healthcare system [3].

A resilient healthcare system is the crux of any emergency preparedness response, with providers building up surge capacity during normal operations in preparation of a high impact occurrence which could escalate healthcare demand and strain existing resources [4, 5]. Transmission risks within the community must be minimized, hence, the role of an enhanced surveillance system is crucial to detect and isolate cases promptly. This would ideally encompass stakeholders from various settings. Previous studies conducted in Singapore after the SARS outbreak in 2003 have highlighted the important role of primary health providers, as many cases sought medical attention from their family physicians as the first level of contact [6, 7]. Furthermore, capitalizing on primary care providers could potentially augment care capacities of the healthcare system in outbreak situations [6, 7]. International studies have also demonstrated the value of primary healthcare providers in sentinel surveillance [5, 8–12].

In Singapore, government and independent private primary care clinics form the stronghold of the primary care clinics. A significant portion of private clinics have been voluntarily engaged under the Public Health Preparedness Clinics scheme [13]. These clinics, deployed in times of public health crisis to provide affordable community care and early detection of suspected cases, were activated in February 2020 in response to the COVID-19 situation [14].

As health-seeking behavior is a critical factor to take into consideration in emergency preparedness models, this study aims to describe the characteristics of laboratory confirmed COVID-19 cases and their health-seeking behavior prior to hospital admission and isolation. The findings provide insights into reviewing and tailoring public health messaging to guide appropriate health-seeking behavior during the period of an infectious disease outbreak.

## Methods

We included all cases diagnosed with COVID-19 by SARS-CoV-2 real-time polymerase chain reaction in Singapore from 23 January to 17 March 2020, whose data was collected as part of routine epidemiological investigations under the Infectious Diseases Act. We excluded (i) asymptomatic cases as they were unlikely to visit primary healthcare institutions, (ii) dormitory cases as this subpopulation had different health-seeking behavior and diagnosis workflow, and (iii) persons subsequently determined to be false positives. As we collected details on visits prior to hospitalization for cases reported until 17 March 2020, the study period was confined to about 1.8 months from the report of the first diagnosed case of the COVID-19 outbreak.

Primary care was defined as community ambulatory health services, consisting of private general practitioner clinics and government-subsidized public clinics, in addition to emergency department (ED) of hospitals where patients were not admitted. Locally-acquired cases were patients who did not report travelling outside Singapore up to 14 days before symptoms onset. Imported cases were travellers who returned to Singapore within 14 days of symptom onset.

We compared health-seeking behavior in different subgroups of COVID-19 cases: (a) imported vs. locally-acquired, and (b) those who sought care from one primary care provider vs. multiple care providers within the group of locally-acquired cases. Variables of interest included number of primary healthcare visits prior to hospitalization, timing of first visit, duration from symptom onset to admission, and medical touchpoints where cases reported at least one visit.

Numbers and proportions were presented for categorical variables, and median and interquartile range (IQR) for continuous variables. Fisher’s exact test or Chi-square test was used to compare categorical variables, and Mann–Whitney U test to compare continuous variables between any two groups. We used Spearman rank correlation to measure the association between the number of visits prior to hospitalization and duration from symptom onset to hospital admission. All statistical tests were two-sided, and statistical significance was taken as *p* < 0.05. Statistical analyses were performed using IBM SPSS Statistics for Windows, V.24.0 and figures were generated using R version 3.6.2 (R Foundation for Statistical Computing, Vienna, Austria).

## Results

We included 258 cases in our analysis, after excluding 5 asymptomatic cases, 2 dormitory cases, and 2 who were later determined to be false positives from the initial 267 cases reported from 23 January to 17 March 2020.

### Comparison of locally-acquired and imported cases

Locally-acquired cases were older (median 50 years, IQR 35–61) than imported cases (median 40 years, IQR 31–53) (Table 1). Males constituted a higher proportion of imported cases than locally-acquired cases (64.9% vs 51.6%, *p* = 0.039). A higher percentage of locally-acquired cases were Singapore residents compared with imported cases (85.7% vs 43.3%, *p* < 0.001).

**Table 1:** Characteristics of locally-acquired and imported COVID-19 cases in Singapore, 23 January to 17 March 2020

Characteristics	All	Locally-acquired	Imported	*P*-value^
	**(*N* = 258)**	**(*N* = 161)**	**(*N* = 97)**	
Age in years, median (IQR)	45 (34–57)	50 (35–61)	40 (31–53)	0.005
Male, *n* (%)	146 (56.6)	83 (51.6)	63 (64.9)	0.039
Singapore resident, *n* (%)	180 (69.8)	138 (85.7)	42 (43.3)	< 0.001
Number of visits before admission, median (IQR)	1 (1–2)	1 (1–2)	1 (1–1)	0.009
Number of visits before admission, *n* (%)				< 0.001
0	45 (17.4)	33 (20.5)	12 (12.4)	
1	122 (47.3)	55 (34.2)	67 (69.1)	
2	50 (19.4)	38 (23.6)	12 (12.4)	
3	30 (11.6)	25 (15.5)	5 (5.2)	
≥ 4	11 (4.3)	10 (6.2)	1 (1.0)	
Days from symptom onset to admission, median (IQR)	5 (3–8)	6 (4–9)	4 (2–7)	< 0.001
**Cases with at least 1 visit**	**All cases**	**Locally-acquired**	**Imported**	***P*-value^**
	**(*n* = 213)**	**(*n* = 128)**	**(*n* = 85)**	
At least 1 visit, *n* (%)				
Primary care clinics	163 (76.5)	115 (89.8)	48 (56.5)	< 0.001
ED	87 (40.8)	34 (26.6)	53 (62.4)	< 0.001
Timing of first visit (for those with at least 1 visit), median (IQR)	2 (1–4)	2 (1–3)	2 (1–4)	0.466
Timing of first visit (for those with at least 1 visit), n (%)				0.907
Day 1	61 (28.6)	37 (28.9)	24 (28.2)	
Day 2	67 (31.5)	43 (33.6)	24 (28.2)	
Day 3	30 (14.1)	17 (13.3)	13 (15.3)	
Day 4	21 (9.9)	13 (10.2)	8 (9.4)	
Day 5	14 (6.6)	8 (6.3)	6 (7.1)	
Day 6 or later	20 (9.4)	10 (7.8)	10 (11.8)	

*IQR *interquartile range, *ED* emergency department

^ *P*-value from Fisher’s exact test or Chi-square test was used for categorical variables and Mann–Whitney U test for continuous variables to compare locally-acquired cases and imported cases

213 cases (82.6%) had at least one visit prior to hospitalization, and among these, 60.1% first sought medical attention at primary care clinics or hospital ED prior to admission within two days of symptom onset with no statistical difference detected between locally-acquired and imported cases (62.5% vs 56.5%, *p* = 0.395). Majority (89.8%) of locally-acquired cases attended primary care clinics at least once in their patient journey compared with 56.5% of the imported cases (*p* < 0.001).

Among cases with at least one visit to care providers prior to hospital admission, the proportion with at least one visit to private general practitioner clinics and/or government-subsidized public clinics was higher among Singapore residents than non-residents (84.0% vs. 58.7%, *p* < 0.001). About 10.2% of locally-acquired cases attended ED without having any clinic visits, compared with 43.5% of imported cases (*p* < 0.001).

**Fig. 1:**
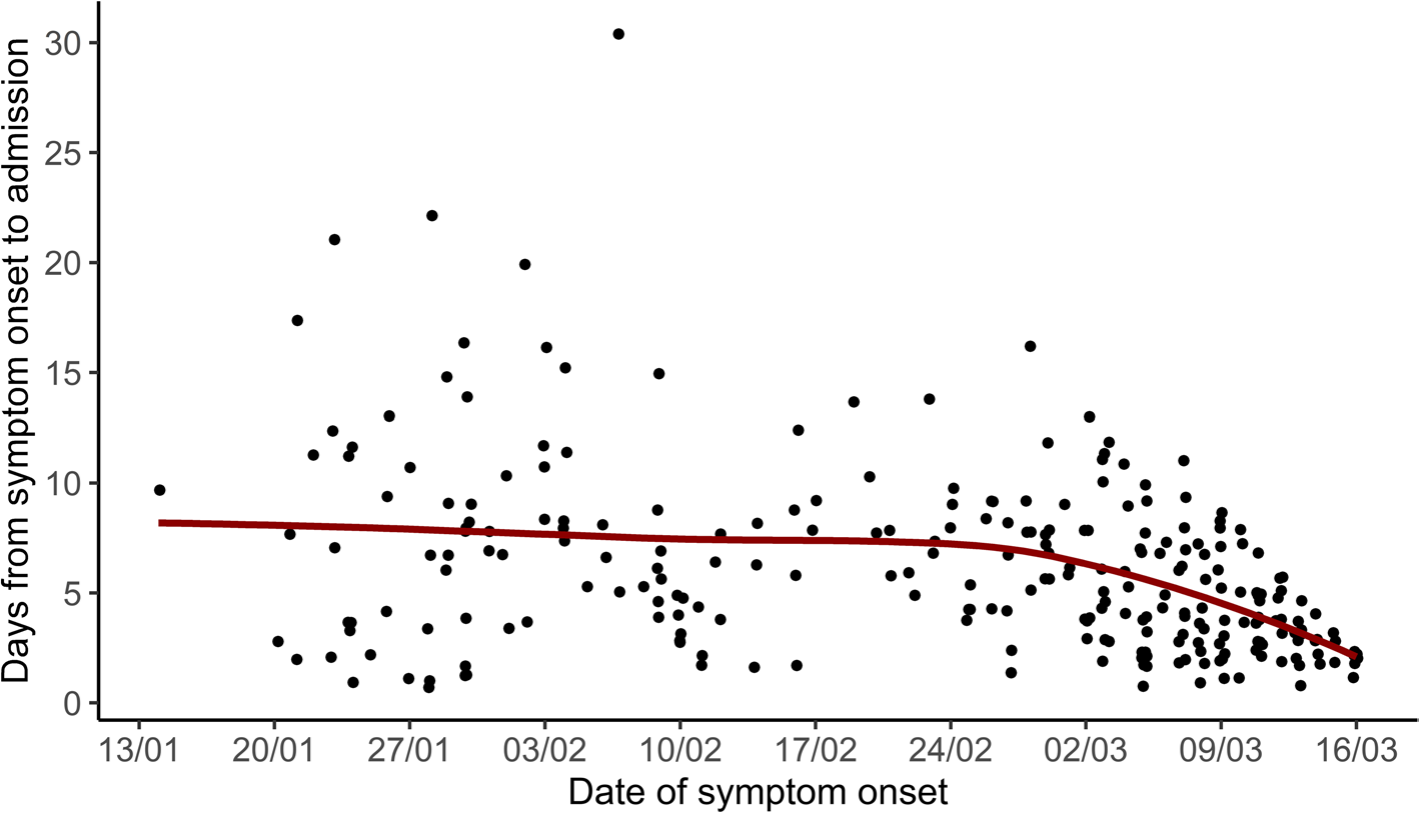
Scatterplot with locally estimated scatterplot smoothing curve of duration from symptom onset to hospital admission and date of symptom onset among COVID-19 cases

Approximately 21.7% of locally-acquired cases had three or more visits to primary care clinics and/or ED compared with 6.2% of imported cases (*p* < 0.001). Locally-acquired cases had longer duration from symptom onset to hospital admission (median 6 days, IQR 4–9) than imported cases (median 4 days, IQR 2–7) (*p* < 0.001). This duration decreased as the epidemic progressed (Fig. 1).

**Fig. 2:**
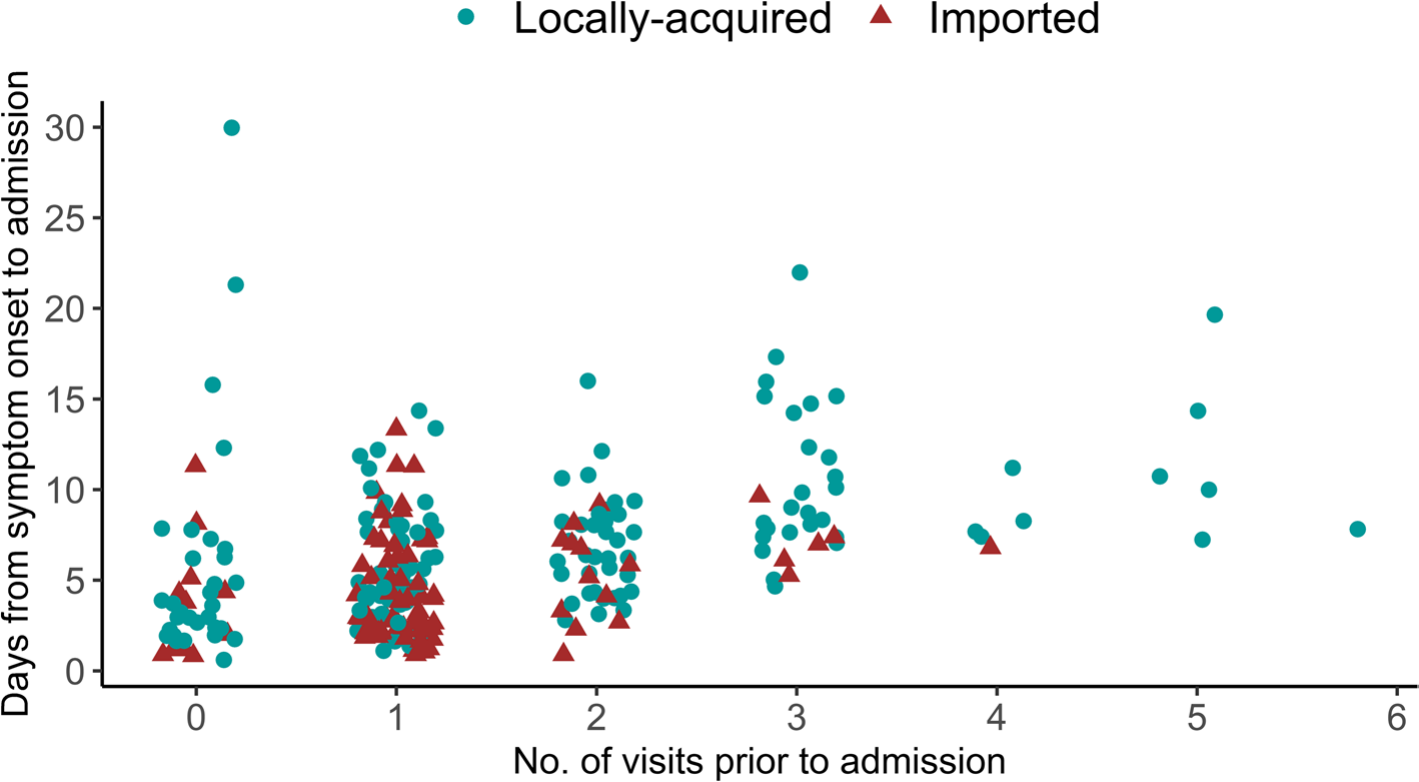
Scatterplot of duration from symptom onset to hospital admission and number of primary care visits before admission among COVID-19 cases

The number of primary care visits and duration from symptom onset to hospital admission exhibited a more widespread distribution among locally-acquired cases (Fig. 2). There was a positive correlation between the number of visits prior to hospitalization and duration from symptom onset to hospital admission (Spearman’s rho = 0.461, *p* < 0.001).

### Comparison of locally-acquired cases who had at least two primary care visits to single care provider and multiple care providers

**Fig. 3:**
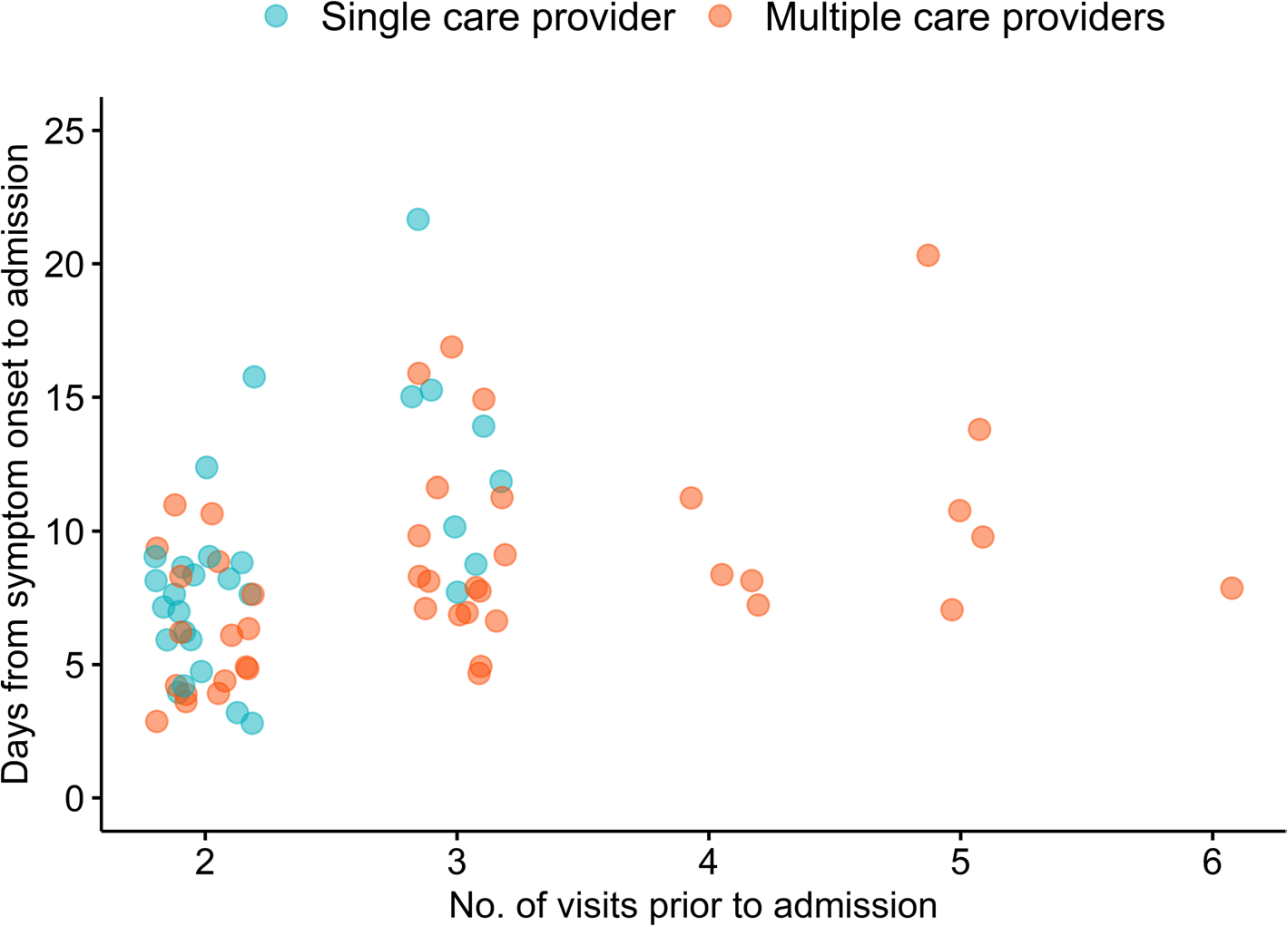
Scatterplot of duration of symptoms and number of primary care visits prior to hospital admission among locally-acquired COVID-19 cases who had two or more visits

Among locally-acquired cases with two or more visits, there was no significant difference between those who sought care from single and multiple care providers in their demographics: age (median 53 vs 48 years, *p* = 0.446), gender (62.1% vs 65.9% were males, *p* = 0.805) and nationality (86.2% vs 93.2% Singapore residents, *p* = 0.425) (Table 2). There were also no differences in timing of first visit, duration from symptom onset to admission and length of hospital stay. Cases who sought care from the same provider had fewer visits compared with those with different care providers (median 2 vs. 3, *p* = 0.001). A higher proportion of cases who saw multiple providers (61.4%) had at least three visits compared with those who saw a single provider (27.6%) (*p* = 0.008). In addition, 72.4% of the latter group had two visits prior to their hospital admission (Fig. 3). In contrast, those with multiple care providers had up to six visits.

**Table 2:** Characteristics of locally-acquired COVID-19 cases who had two or more visits to single care provider and multiple care providers prior to hospital admission

Characteristics	All	Single care provider	Multiple care providers	*P*-value^
	**(*N* = 73)**	**(*N* = 29)**	**(*N* = 44)**	
Age in years, median (IQR)	51 (38–62)	48 (36–61)	53 (39–62)	0.446
Male, *n* (%)	47 (64.4)	18 (62.1)	29 (65.9)	0.805
Singapore resident, *n* (%)	66 (90.4)	25 (86.2)	41 (93.2)	0.425
Number of visits before admission, median (IQR)	2 (2–3)	2 (2–3)	3 (2–3)	0.001
Number of visits before admission (Primary care clinics / ED), *n* (%)				0.026
2	38 (52.1)	21 (72.4)	17 (38.6)	
3	25 (34.2)	8 (27.6)	17 (38.6)	
4	4 (5.5)	0 (0.0)	4 (9.1)	
5	5 (6.8)	0 (0.0)	5 (11.4)	
6	1 (1.4)	0 (0.0)	1 (2.3)	
Timing of first visit, median (IQR)	2 (1–3)	2 (1–3)	2 (1–3)	0.403
Timing of first visit, *n* (%)				0.937
Day 1	22 (30.1)	10 (34.5)	12 (27.3)	
Day 2	27 (37.0)	11 (37.9)	16 (36.4)	
Day 3	12 (16.4)	4 (13.8)	8 (18.2)	
Day 4	6 (8.2)	2 (6.9)	4 (9.1)	
Day 5	5 (6.8)	2 (6.9)	3 (6.8)	
Day 6 or later	1 (1.4)	0 (0.0)	1 (2.3)	
Days from symptom onset to admission, median (IQR)	8 (6–11)	8 (6–11)	8 (6–11)	0.586
Length of hospital stay (days), median (IQR)	15 (10–22)	17 (11–27)	15 (10–20)	0.429
Ever admitted to ICU, *n* (%)	17 (23.3)	6 (20.7)	11 (25.0)	0.781

*ICU *intensive care unit, *IQR *interquartile range

^ *P*-value from Fisher’s exact test or Chi-square test was used for categorical variables and Mann–Whitney U test for continuous variables to compare single care provider and multiple care providers

## Discussion

Our study revealed differences in health-seeking behavior among subgroups of the initial COVID-19 cases in Singapore. Locally-acquired cases had significantly longer duration from symptom onset to hospital admission than imported cases (median 6 days vs. 4 days). Among those with at least one visit prior to admission, a higher percentage of locally-acquired cases had sought medical attention at the primary care level than imported cases (89.8% vs. 56.5%).

Imported cases were more likely to attend ED, bypassing primary care clinics completely (43.5% of imported cases vs 10.2% of locally-acquired cases attended ED directly without any clinic visits, *p* < 0.001), and those who attended clinics had fewer visits before being referred for further testing at the national screening centre or other EDs (6.2% of imported cases had at least three primary care visits prior to admission compared with 21.3% of locally-acquired cases) (Table 1). This observation could be due to two reasons: first, imported cases consisted mostly of Singapore international undergraduate students and foreigners working in Singapore, who were less likely to have a regular physician and with a raised perception of their infection risk, would attend ED directly or earlier in their patient journey; second, the heightened vigilance of primary care physicians towards this group. As the pandemic unfolded and more was known about the virus, Singapore Ministry of Health issued travel advisories and revised circulars circulated to doctors on suspect case definitions. Hence, the attitude of doctors would evolve accordingly with a lower threshold on testing patients deemed to be at increased risk of infection, which resulted in fewer visits by imported cases.

In contrast, doctors’ perception of lower community transmission risk at that time resulted in locally-acquired cases being referred only after lack of clinical improvement despite repeated visits. Equally crucial is the patient’s personal cognizance and health literacy [15–17]. Locally-acquired cases might have attributed their symptoms to a common cold or gastroenteritis, resulting in delays lasting up to a month in seeking medical attention. While seemingly innocuous under normal circumstances, this could have devastating consequences in an outbreak situation.

Overall, Singapore residents were more likely to attend primary care clinics than non-residents (84.0% vs 58.7%). Fever, cough, sore throat and diarrhoea were common presenting symptoms of COVID-19 cases [18], routinely managed within the community by family physicians.

Among the subgroup of locally-acquired cases with at least two primary care visits who saw the same care provider (median of 2 visits), a smaller proportion (27.6%) had three or more visits before being referred and admitted, compared with 61.4% of those who saw different providers (median of 3 visits). Having no basis for comparison from previous visits, a different healthcare provider lacks pertinent information when formulating management plan for the patient, which typically leads to later diagnosis, isolation and treatment, and consequently increases transmission risk. Our results thus highlight the risk of seeking care from multiple care providers or “doctor shopping” within the same episode of illness [19–21].

Doctor shopping could be attributed to various factors. One factor is healthcare accessibility; with high concentrations of primary care clinics island-wide, the convenience of attending clinics near one’s workplace and home outweighs care continuity concerns [19, 22–24]. Another factor is unmet expectations; patients might have misconceptions of partaking less efficacious medications as symptoms persist, or feel unsatisfied with previous consultations [19, 22–24]. Hence, appropriate public health communication to the public is crucial even during peacetime.

We acknowledged several limitations in our study. The observational design of our study precluded causal inference. This study was limited to the initial COVID-19 period where cases were predominantly imported. As the epidemic progressed, health-seeking behavior would evolve and as such, an in-depth study would be useful to ascertain attitudes and responses of the Singapore population at each phase of the outbreak. Our study was confined to cases diagnosed and managed in Singapore, and the findings may not be generalized to health-seeking behavior of COVID-19 cases in other countries with different health systems and financing mechanisms. As some information related to primary healthcare visits prior to hospitalization was ascertained based on self-reporting, the data collected was subjected to recall bias. Nevertheless, there were standard operating procedures in place to ensure the accuracy and consistency of information documented, such as having trained public health officers to interview the cases, and verifying movements reported by the cases from other sources.

## Conclusion

Our findings demonstrated high attendance rates of cases within primary care settings, especially for Singapore residents, underscoring the significance of these care providers as trusted sources for patients. Thus, policy makers could consider creating a comprehensive sentinel surveillance network comprising private and public primary care clinics, which would enable early detection of suspected cases within the community.

Among the locally-acquired cases, our results favour the proposition of every resident seeking early medical attention from a single care provider, which would facilitate early detection and isolation, hence limiting local transmission and enabling better control of the pandemic. This demonstrates the importance of population health literacy especially during an outbreak, where there is a need for individuals to adopt appropriate healthcare seeking behavior (i.e. recognize when they should seek medical advice and visit the same care provider) and for the authorities to communicate public health messages simply and promptly via trusted channels [16, 17].

Our study did not delve into population healthcare literacy and specific reasons for health-seeking behavior, which could constitute future considerations to inform and enhance public health prevention strategies.

## Data Availability

The data that support the findings of this study are available from the Ministry of Health, Singapore but restrictions apply to the availability of these data, which were used under license for the current study, and so are not publicly available. However, if requested, Dr Matthias Paul HS Toh can assess the reasonableness and seek the approval of the Ministry of Health for approval to release the data.

## Abbreviations

ED: Emergency department
IQR: Interquartile range
SARS-CoV-2: Severe acute respiratory syndrome coronavirus 2
